# The *DISC1* R264Q variant increases affinity for the dopamine D2 receptor and increases GSK3 activity

**DOI:** 10.1186/s13041-020-00625-1

**Published:** 2020-06-03

**Authors:** Ping Su, Hailong Zhang, Albert H. C. Wong, Fang Liu

**Affiliations:** 1 Campbell Family Mental Health Research Institute, Centre for Addiction and Mental Health, Toronto, Ontario M5T 1R8 Canada; 2 Institute of Medical Science, University of Toronto, Toronto, Ontario Canada; 3 Department of Psychiatry, University of Toronto, Toronto, Ontario Canada; 4 Department of Physiology, University of Toronto, Toronto, Ontario Canada

**Keywords:** Dopamine D2 receptor (D2R), Disrupted in schizophrenia 1 (DISC1), Glycogen synthase kinase (GSK) -3, Schizophrenia

## Abstract

The *Disrupted in schizophrenia 1* (*DISC1*) gene encodes a scaffolding protein that is involved in many neural functions such as neurogenesis, neural differentiation, embryonic neuron migration and neurotransmitter signalling. *DISC1* was originally implicated in schizophrenia in a single family with a drastic mutation, a chromosomal translocation severing the mid-point of the gene (aa 598). Some common *DISC1* variants have also been associated with schizophrenia in the general population, but those located far from the chromosomal translocation breakpoint likely have a different functional impact. We previously reported that DISC1 forms a protein complex with dopamine D2 receptor (D2R), the main target for antipsychotic medications. The D2R-DISC1 complex is elevated in brain tissue from schizophrenia patients and facilitates glycogen synthase kinase (GSK)-3 signaling. The *DISC1* R264Q variant is located within the region that binds the D2R, and we found that this polymorphism increases the affinity of DISC1 for the D2R and promotes GSK3 activity. Our results suggest a possible mechanism by which this common polymorphism could affect aspects of brain function that are relevant to psychosis and schizophrenia. This provides additional insight into molecular mechanisms underlying schizophrenia that could be exploited in the development of novel pharmacological treatments.

## Introduction

*Disrupted in schizophrenia 1 (DISC1)* is an important susceptibility gene for many psychiatric disorders because it codes for a powerful regulatory protein with a large interacting network that regulates fundamental brain functions [1]. The *DISC1* gene was originally discovered in a single large family carrying a chromosomal translocation that severs *DISC1* roughly in half [2, 3]. Although common *DISC1* variants are not the strongest associations with schizophrenia in genome-wide association study (GWAS), the drastic phenotype in the *DISC1* translocation family and in *Disc1* mutant animal models provides a useful entry point to understand the pathobiology of psychiatric symptoms and potential disease mechanisms [4, 5].

Our group previously discovered that the DISC1 protein forms a protein-protein complex with the dopamine D2 receptor (D2R), the main target of all existing antipsychotic medications [6]. We found that the DISC1-D2R complex is elevated in post-mortem brain samples from patients with schizophrenia, and in *Disc1*-L100P mutant mice, an animal model for schizophrenia. The complex facilitates glycogen synthase kinase (GSK)-3 signaling and inhibits agonist-induced D2R internalization. Disrupting the complex with an interfering peptide can reverse abnormal behaviours relevant to schizophrenia in *Disc1*-L100P mice. Thus, we hypothesized that the common *DISC1* gene variant R264Q *DISC1*, located within the binding region with the D2R could alter the strength of the DISC1-D2R interaction and have functional consequences related to the pathophysiology of schizophrenia. The *DISC1* R264Q variant has previously been associated with schizophrenia [7], and has been reported to impair GSK-3 signaling and neurodevelopment [8].

The D2 receptor is one of five dopamine receptors that are all G-protein coupled transmembrane monomeric receptors, each encoded by a single discrete gene [9]. The D2, D3 and D4 receptors couple to Gi/o and thereby inhibit adenylyl cyclase, while the D1 and D5 receptors have the opposite functional effect by coupling with Gs to activate adenylyl cyclase [10]. All established antipsychotic medications target the dopamine D2 receptor and thus it is one of the most robust modulators of psychotic symptoms [11]. GSK3 is a hub protein on which numerous signal paths converge, including Wnt [12], insulin [13], Trk [14], and several subtypes of dopamine and serotonin receptors [15]. Many antipsychotics inhibit GSK3 through increased serine phosphorylation [16, 17], and so does lithium [17–19], which is the oldest and still most effective prophylactic medication for bipolar disorder [20, 21]. Thus, we sought to discover additional mechanistic links between DISC1 and these other known regulators of psychosis by investigating the functional impact of a schizophrenia-associated *DISC1* variant located within the region that binds the D2 receptor.

## Materials and methods

### Drugs

Quinpirole was purchased from Sigma-Aldrich, and was freshly prepared every time before treatment by dissolving into distilled water with a concentration of 10 mM.

### GST fusion protein constructs and DNA subcloning

GST-fusion proteins encoding N-terminus of DISC1 were amplified by PCR from full-length human or mouse cDNA clones. All constructs were sequenced to confirm the absence of spurious PCR-generated nucleotide errors. GST-fusion proteins were prepared from bacterial lysates with Glutathione Sepharose 4B beads as instructed by the manufacturer (Amersham) as previously described. To construct GST-fusion proteins encoding DISC1_NT_, cDNA fragments were amplified by PCR with specific primers, and subcloned into pGEX-4 T-3 vector. All constructs were re-sequenced to confirm appropriate splice fusion and the absence of spurious PCR generated nucleotide errors.

### Plasmid mutation

Mutants of GST-DISC1_NT_ and Flag-DISC1 were created with the QuickChange site-directd mutagenesis kit (Stratagene). All mutants were confirmed by DNA sequencing.

### Cell culture and DNA transfection

HEK293T cells were maintained at 37 °C in Dulbecco’s Modified Eagle Medium (DMEM) (Gibco) supplemented with 10% fetal bovine serum (Gibco). Cells were grown to 90% confluency before being transiently transfected with DNA constructs using Xtreme gene 9 transfection reagent (Roche), following the manufacturer’s instruction. Cells were used for various experiments after 48 h of transfection.

### Co-immunoprecipitation, protein affinity purification and western blot

Co-immunoprecipitation and western blot assays were conducted as previously described [6, 22, 23]. For co-immunoprecipitation experiments, 500–700 μg solubilized protein extracted from HEK293T cells (ATCC) was incubated in the presence of primary anti-HA antibody (Roche) or IgG (negative control) (1–2 μg) together with protein A/G plus agarose (Santa Cruz Biotechnology) at 4 °C for 12 h. Pellets were washed, boiled for 5 min in SDS sample buffer (Bio-Rad) and subjected to SDS-PAGE. 50–100 μg of protein extracted from HEK293T cells was used as a control in each experiment. For affinity purification experiments, 500 μg of protein extracted from mouse striatum was incubated with glutathione-sepharose beads (Amersham) bound to the indicated GST-fusion proteins (50–150 μg) at 4 °C for 12 h. Beads were washed, boiled for 5 min in SDS sample buffer and subjected to SDS-PAGE. After transfer of proteins onto nitrocellulose, membranes were western blotted with the primary antibodies: anti-HA (1:1000, rat, Roche), anti-Flag (1:2000, mouse, Sigma-Aldrich), anti-D2R (1:200, mouse, Santa Cruz Biotechnology). The intensity of protein level was quantified by densitometry (software: ImageLab, Bio-Rad). For detection of phosphorylation of GSK3, the antibodies used include: anti-pGSK-3α/β (S21/S9) (Cell Signaling Technology, rabbit), anti-GSK-3α (Cell Signaling Technology, rabbit), anti-GSK-3β (Cell Signaling Technology, rabbit).

### Surface Plasmon resonance (SPR) spectroscopy

All SPR experiments were performed on a SR7500DC (Reichert, USA) at 25 °C in the running buffer TBS-T, which contains 150 mM NaCl, 1 mM CaCl2, 1 mM MgCl2, 10 mM Tris, pH 7.4, and 0.005% Surfactant P-20. TAT-D2pep peptide (10 μM) were covalently coupled via amine groups onto the carboxymethylated dextran surface sensor chips (Reichert, USA) using 26 EDC/Sulfo-NHS crosslinking strategy. During coupling, TAT-D2pep peptide was injected in 10 mM sodium formate (pH 5.0) to reach 600 Response Units (RU). A flow rate 50 μl/min was set to perform the binding and kinetic analysis in case of the effect of mass transportation limitation. Binding affinity was measured using a range of concentrations from 0 μM to 4.0 μM for GST-DISC1_NT_, respectively. For mouse GST-*Disc1*-L100P_NT_ and GST-DISC1_NT_, binding affinity was measured using a range of concentrations from 25 nM to 400 nM for GST-*Disc1*-L100P_NT_, 100 nM to 1.6 μM for GST-DISC1_NT_, respectively. The duration of the association and dissociation phases was set to 180 s, 300 s, respectively. Regeneration of the sensor chip was achieved by adding 10 mM NaOH, 1 M NaCl. Flow rate was set to 50 uL/min to reduce the effect of mass transport limitations. GST alone was injected as a negative control in the same condition. Data were assembled and analyzed using Scrubber and Clamp XP software packages (University of Utah). All SPR were tested for mass transfer to make sure that curves observed were not limited by mass transfer (data not shown). Equilibrium analysis was conducted for GST-DISC1_NT_ based on the binding affinity of the interactions.

### Statistical analysis

Depending on the experiment, data were analyzed by t-test, one-way analysis of variance (*ANOVA*) followed by Tukey’s post hoc test or two-way *ANOVA* followed by Bonferroni’s post hoc test (Prism 6, GraphPad Software). All data were expressed as mean ± standard error of mean (SEM). The highest significance level of *p* < 0.05 was used for all analyses.

## Results

### *DISC1* variant facilitates DISC1-D2R complex formation

**Fig. 1:**
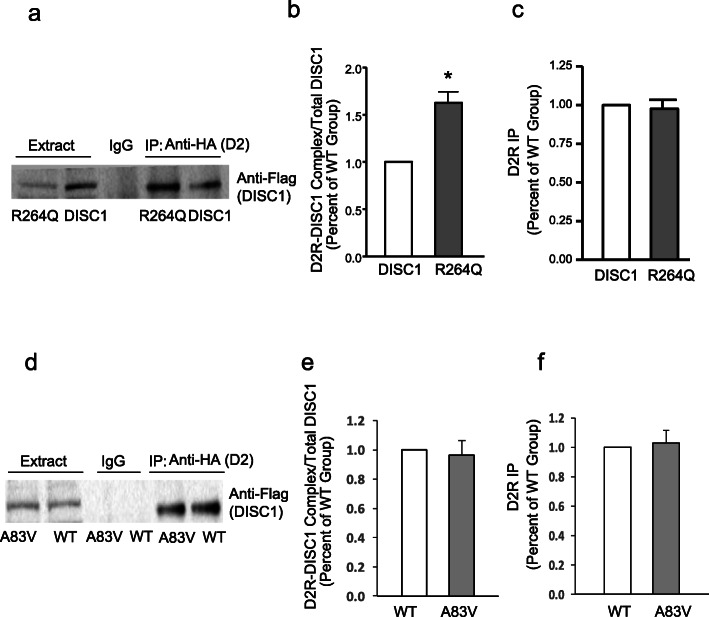
The R264Q *DISC1* variant facilitates the DISC1-D2R complex formation. **a**. DISC1-D2R complex formation is significantly increased in HEK-293 T cells expressing HA-D2R/Flag-DISC1_R264Q_ (R264Q) compared to HEK-293 T cells expressing HA-D2R/Flag-DISC1 (DISC1). Cell lysate was subjected to immunoprecipitation with anti-HA or IgG and immunoblotted with anti-Flag or anti-HA antibodies. **b-c**. Densitometric analysis of precipitated DISC1 or D2R. The intensity of DISC1 or D2R was quantified by densitometry (software: ImageLab, Bio-Rad). *p < 0.05. **d**. DISC1-D2R complex formation does not change in HEK-293 T cells expressing HA-D2R/Flag-DISC1_A83V_ (A83V) compared to HEK-293 T cells expressing HA-D2R/Flag-DISC1 (WT). Cell lysate was subjected to immunoprecipitation with anti-HA or IgG and immunoblotted with anti-Flag or anti-HA antibodies. **e-f**. Densitometric analysis of precipitated DISC1 or D2R. The intensity of DISC1 or D2R was quantified by densitometry (software: ImageLab, Bio-Rad). Data was shown as mean ± SEM, and was analyzed by t-test. *n* = 3

Because some common *DISC1* variants have been associated with schizophrenia [24], we investigated possible functional effects on DISC1-D2R complex formation and GSK3 α/β Ser21/9 phosphorylation. We focused on the R264Q *DISC1* variant because the mutation is within the DISC1 region that interacts with D2R [6]. As shown in Fig. 1a, we found that the human R264Q *DISC1* variant, but not the control A83V variant located outside the binding domain, is associated with significantly lower DISC1 protein levels compared to wildtype (WT) DISC1, when transfected into HEK-293 T cells. However, DISC1-D2R complex levels are significantly increased in DISC1_R264Q_ (Fig. 1a-c), but not DISC1_A83V_ (Fig. 1d-f)_,_ cells as shown by co-immunoprecipitation, despite the decreased expression of DISC1_R264Q_ protein. The levels of directly-immunoprecipitated D2R were not significantly different between the two DISC1 variants.

### *DISC1* variant has a higher affinity for D2R

**Fig. 2:**
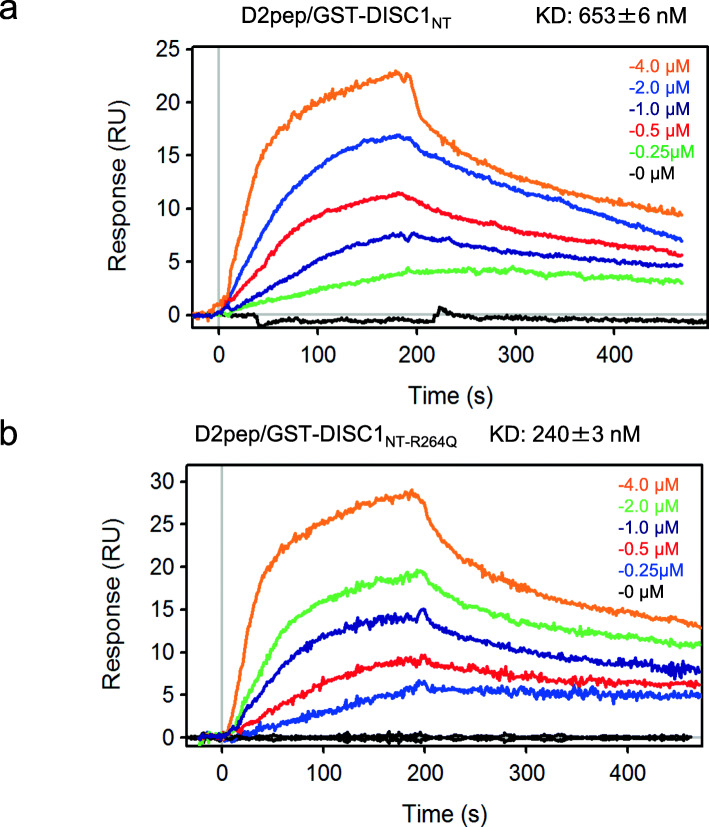
The R264Q *DISC1* variant enhances the binding affinity between DISC1 and D2R. Surface Plasmon Resonance (SPR) analysis of the concentration-dependent binding of GST-DISC1_NT_ (**a**) or GST-DISC1_NT-R264Q_ (**b**) with immobilized TAT-D2pep[K_211_-T_225_] peptide. Binding affinity was measured using a range of concentrations from 0 nM to 4000 nM. The duration of the association and dissociation phases was set to 180 s, 300 s, respectively. Flow rate was set to 50 uL/min to reduce the effect of mass transport limitations. Regeneration of the sensor chip was achieved by adding 10 mM NaOH, 1 M NaCl. Equilibrium analysis was conducted for GST-DISC1_NT_ based on the binding affinity of the interactions

To investigate whether the R264Q mutation can alter the binding affinity between DISC1 and D2R, we measured the affinity between D2pep[K_211_-T_225_] and GST-DISC1-NT_R264Q_ or GST-DISC1-NT using surface plasmon resonance (SPR). D2 [K_211_-T_225_] is the region interacting with DISC1 as previously reported. Our data show that GST-DISC1-NT has a K_D_ value of 653 ± 6 nM (Fig. 2a), while GST-DISC1_R264Q_ has a much higher affinity with a K_D_ value of 243 ± 3 nM (Fig. 2b). Thus, the R264Q *DISC1* variant has a higher binding affinity for D2R, which could account for the greater DISC1-D2R complex levels.

### The DISC1_R264Q_ variant decreases GSK3 α/β Ser-21/9 phosphorylation

**Fig. 3:**
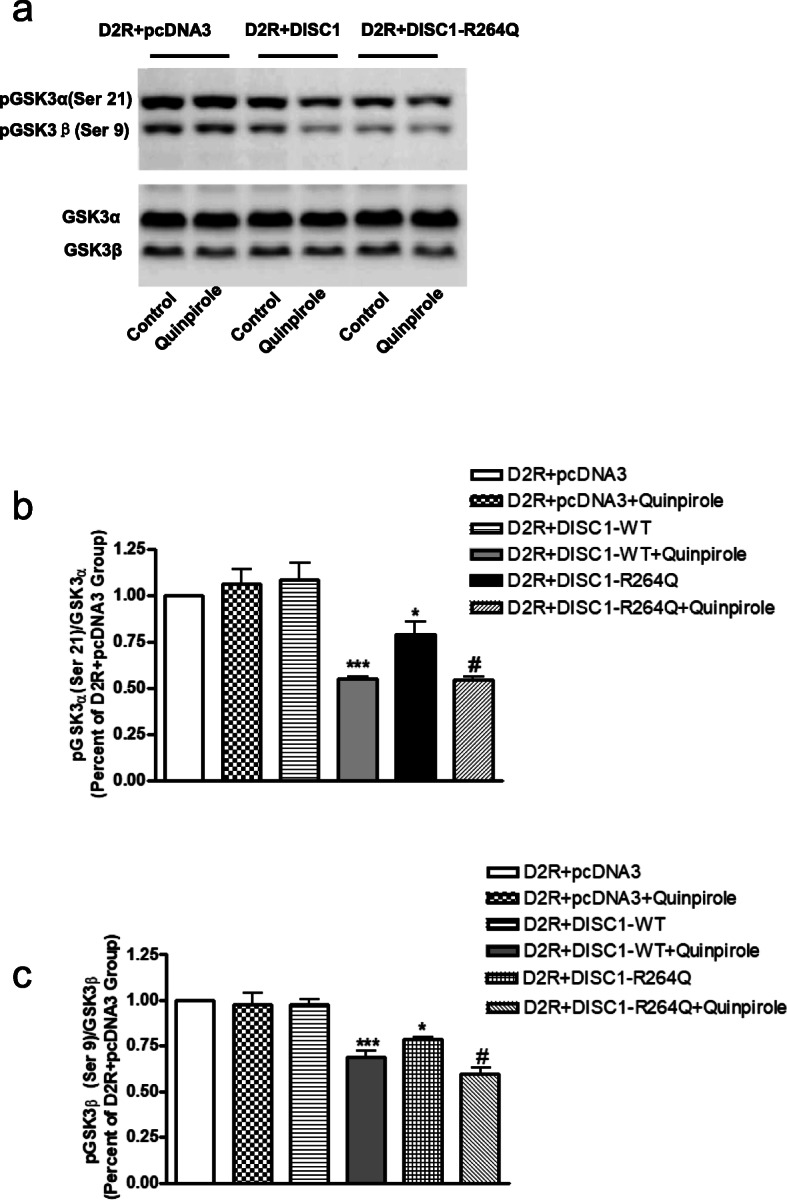
The DISC1_R264Q_ variant decreases GSK3 α/β Ser21/9 phosphorylation. **a.** Activation of D2R reduces GSK-3α/β (Ser21/9) phosphorylation in HEK-293 T cells expressing D2R and DISC1, D2R and *DISC1*-R264Q, but not in HEK-293 cells expressing D2R with pcDNA3, the mammalian expression vector in which DISC1 is sub-cloned. Western blot analysis of phosphorylated GSK-3α/β levels in extract prepared from HEK 293 T cells transfected with D2R with pcDNA3 or D2R with DISC1 or D2R and *DISC1*-R264Q in the presence or absence of quinpirole (10 µM, 30 min). GSK-3α/β was used as a loading control. **b-c.** Densitometric analysis of phosphorylated GSK-3α/β (Ser21/9). The intensity of phospho-GSK-3α/β (Ser21/9) was quantified by densitometry (software: ImageLab, Bio-Rad). Data were analyzed by one-way ANOVA followed by Tukey’s post hoc test. (*p < 0.05, ***p < 0.001, compared to D2R + DISC1-WT group, #*p* < 0.05 as compared to D2R + DISC1-R264Q, *n* = 6). Data was shown as mean ± SEM

We found that the basal level of GSK3 α/β Ser21/9 phosphorylation in R264Q *DISC1* cells is significantly lower than that in cells expressing WT DISC1. Although quinpirole stimulation still reduces GSK3 α/β Ser21/9 phosphorylation in R264Q *DISC1* cells, the percent reduction is smaller than that in wild-type DISC1 expressing cells (Fig. 3a-c). Thus, either activation of D2R or the R264Q *DISC1* mutation can both enhance DISC1-D2R complex formation and decreases GSK3 α/β Ser21/9 phosphorylation.

### The mouse *DISC1*-L100P mutation facilitates DISC1-D2R complex formation

Many different mutant *Disc1* mouse models have shown the important role of this gene in neurodevelopment, neurotransmitter signaling and behaviours relevant to neuropsychiatric disease [25–28]. One of the earliest such models was generated through ENU mutagenesis, and was a point mutation resulting in a single amino-acid substitution, L100P [26]. This mouse line displayed a variety of schizophrenia-related abnormalities including hyperlocomotion, impaired pre-pulse inhibition/latent inhibition, deficient neurogenesis/neuron migration, enhanced dopamine function and dendritic spine deficits rescued by genetic inactivation of GSK3α [26, 29, 30]. Furthermore, in our previous study, we have found that the level of D2R-DISC1 complex is elevated and the GSK3 α/β Ser21/9 phosphorylation is decreased in the *DISC1*-L100P mice [6].

**Fig. 4:**
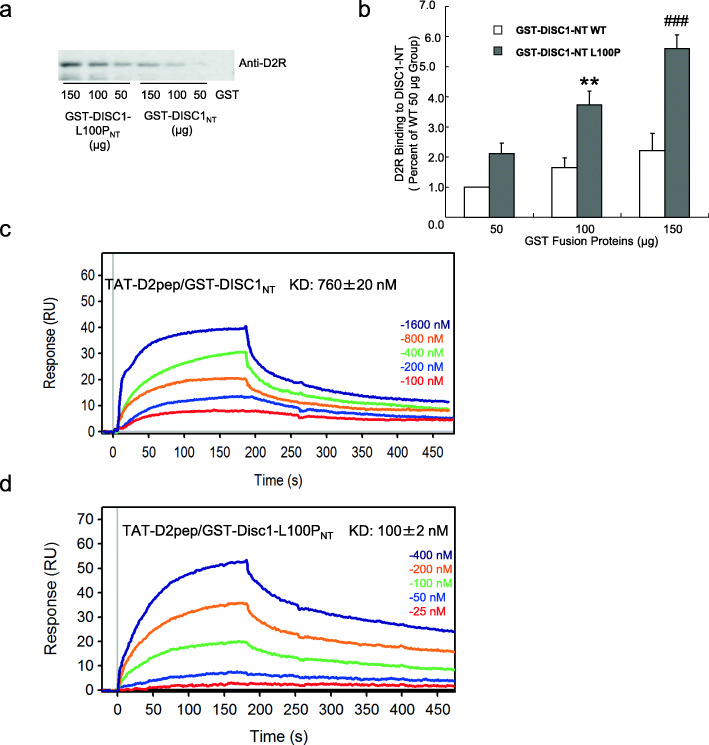
The mouse *DISC1*-L100P mutation increases the binding affinity between DISC1 and D2R. **a.** GST-DISC1_NT_ and GST-*Disc1*-L100P_NT_ affinity “pull-down” of D2R from mouse striatal tissue. Various concentrations (50, 100, 150 μg) of GST-DISC1_NT_ or GST-*Disc1*-L100P_NT_ were incubated with mouse striatal extract and the precipitated proteins were immunoblotted with anti-D2R. **b.** Densitometric analysis of precipitated D2R. The intensity of D2R was quantified by densitometry (software: ImageLab, Bio-Rad). GST-DISC1_NT_ compared with GST-*Disc1*-L100P_NT_ by two-way ANOVA followed by Bonferroni post hoc test (**p < 0.01 as compared to that of 100 μg GST-DISC1_NT_, ###*p* < 0.001 as compared to that of 150 μg GST-DISC1_NT_, *n* = 3). Data was shown as mean ± SEM. **c-d**. Surface Plasmon Resonance (SPR) analysis of the concentration-dependent binding of GST-DISC1_NT_ (**c**) or GST-*Disc1*-L100P_NT_**(d)** with immobilized TAT-D2pep[K211-T225] peptide. Binding affinity was measured using a range of concentrations from 25 nM to 400 nM for GST-*Disc1*-L100P_NT_, and 100 nM to 1.6 μM for GST-DISC1_NT_, respectively. The duration of the association and dissociation phases was set to 180 s, 300 s, respectively. Flow rate was set to 50 uL/min to reduce the effect of mass transport limitations. Regeneration of the sensor chip was achieved by adding 10 mM NaOH, 1 M NaCl

The Disc1-L100P mutation is located within the Disc1 interacting site with the D2R, we further tested the Disc1-L100P mutation could affect the binding affinity between the two proteins. We confirmed this hypothesis with two different methods: affinity pull-down assay and surface plasmon resonance (SPR). We found that GST-*Disc1*-L100P_NT_ is able to “pull down” much higher amounts of D2R compared to GST-DISC1_NT_ (Fig. 4a-b)_._ SPR analysis of affinity between TAT-D2pep[K_211_-T_225_] peptides and GST-*Disc1*-L100P_NT_ or GST-DISC1_NT_ allowed estimation of association and dissociation rates corresponding to a K_D_ value of 760 ± 20 nM for GST-DISC1_NT_, while GST-*Disc1*-L100P_NT_ had a 7.6-fold higher affinity with a K_D_ value of 100 ± 2 nM (Fig. 4c-d). Thus, similar to the R264Q *DISC1* variant, the *Disc1*-L100P mutation has a higher binding affinity for D2R, which could account for the greater DISC1-D2R complex levels observed in our previous study.

## Discussion

In summary, we found that the *DISC1* R264Q variant has higher binding affinity for the dopamine D2 receptor, and results in higher levels of the DISC1-D2R protein complex in conjunction with decreased GSK3α/β Ser21/9 phosphorylation. These results suggest possible molecular mechanisms contributing to schizophrenia because the DISC1-D2R complex is higher in patients with schizophrenia [6] and the *DISC1* R264Q variant has been associated with schizophrenia [7]. Similar results were seen with the *Disc1*-L100P mutation in the mouse, which has a similar effect to the *DISC1* R264Q variant. These data converge with the accumulating evidence for the important role of DISC1 in the pathophysiology of schizophrenia and other major psychiatric disorders.

The *DISC1* R264Q variant has previously been shown to disrupt Wnt/GSK3β signaling and brain development [8], through decreased DISC1 R264Q binding to GSK3β. That group also found that DISC1 R264Q increases GSK3β Y216 phosphorylation, leading to higher GSK3β activation and reducing Wnt signaling, all in human-derived lymphoblast cell lines. They did not observe changes in GSK3β Serine 9 phosphorylation. In contrast, our experiments used transfected HEK293T cells and we found phosphorylation of Serine 21 and 9 to be reduced in cells transfected with *DISC1* R264Q compared with wild type *DISC1*. An important difference between our experimental designs was our use of the dopamine agonist quinpirole to activate the D2 receptor. However, there were still differences in our data at baseline, without agonist stimulation, that could be due to differences in the cell model system used.

Another possible factor that could cause these divergent results is that Singh et al. studied lymphoblasts from patients homozygous for one of the two *DISC1* R264Q alleles [8]. These cell lines were obtained from a NIMH sample of bipolar disorder and had been transformed using Epstein-Barr virus. Thus, there are numerous possible biological differences between our transfected HEK293T cells and the transformed human lymphoblastoid cell lines that could lead to differences in the observed baseline GSK3β Serine 9 phosphorylation. Finally, we used an antibody against phosphorylated GSK-3α/β Ser21/9 (Cell Signaling Technology, rabbit), while Singh et al. did not specify the antibody used to detect GSK3β Serine 9 phosphorylation. It is possible that there are significant differences only in GSK3α Serine 21 that account for the apparent discrepancy between our two observations.

The significance our findings is to link human genetic associations between a common *DISC1* gene variant and functional molecular biological changes that could contribute to the pathophysiology of schizophrenia. Research on DISC1 and psychiatric disorders has generated many new insights into the origins of schizophrenia, especially the role of DISC1 in cortical neurogenesis, neuroblast migration and early development [8, 31, 32]. The DISC1-D2R protein complex and enhancement by the R264Q variant provides additional insights into schizophrenia biology because we found previously that schizophrenia patients in general have higher DISC1-D2R complex levels. The increased DISC1-D2R complex levels with DISC1 R264Q provides further evidence for the mechanism by which schizophrenia-associated DISC1 variants can increase disease susceptibility. We also proposed that disrupting the DISC1-D2R complex with a therapeutic peptide or small molecule drugs could be a novel treatment strategy for schizophrenia. Identifying genetic markers for patients with increased DISC1-D2R complex levels could allow treatments to be personalized and focused on patients most likely to benefit.

## Data Availability

The datasets used and/or analyzed during the current study are available from the corresponding author on reasonable request.

## Abbreviations

D2R: Dopamine D2 receptor
DISC1: Disrupted in schizophrenia 1
GSK-3: Glycogen synthase kinase − 3
SPR: Surface plasmon resonance
GST: Glutathione S-transferase
TAT: Trans-activator of transcription
GWAS: Genome-wide association study
